# The Production Possibility of the Antimicrobial Filaments by Co-Extrusion of the PLA Pellet with Chitosan Powder for FDM 3D Printing Technology

**DOI:** 10.3390/polym11111893

**Published:** 2019-11-16

**Authors:** Szymon Mania, Jacek Ryl, Jia-Rong Jinn, Ya-Jane Wang, Anna Michałowska, Robert Tylingo

**Affiliations:** 1 Chemical Faculty, Department of Chemistry, Technology and Biotechnology of Food, Gdansk University of Technology, 11/12 G. Narutowicza Str., 80-233 Gdansk, Poland; robertt@pg.edu.pl; 2 Chemical Faculty, Department of Electrochemistry, Corrosion and Material Engineering, Gdansk University of Technology, 11/12 G. Narutowicza Str., 80-233 Gdansk, Poland; jacek.ryl@pg.edu.pl; 3 Department of Food Science, University of Arkansas, 2650 N. Young Ave., Fayetteville, AR 72704, USA, yjwang@uark.edu (Y.-J.W.); 4 AGC Biologics, Vandtårnsvej 83B, 2860 Søborg, Copenhagen, Denmark; anna.s.michalowska@gmail.com

**Keywords:** antimicrobial properties, chitosan, FDM, PLA, sPLA, two-component filament, 3D printing

## Abstract

The last decades have witnessed a major advancement and development in three-dimensional (3D) printing technology. In the future, the trend’s utilization of 3D printing is expected to play an important role in the biomedical field. This work presents co-extrusion of the polylactic acid (PLA), its derivatives (sPLA), and chitosan with the aim of achieving filaments for printing 3D objects, such as biomedical tools or implants. The physicochemical and antimicrobial properties were evaluated using SEM, FT-IR, DSC, instrumental mechanical test, and based on the ASTM E2149 standard, respectively. The addition of chitosan in the PLA and sPLA filaments increased their porosity and decreased density. The FT-IR analysis showed that PLA and chitosan only formed a physical mixture after extrusion. The addition of chitosan caused deterioration of the mechanical properties of filaments, especially elongation at break and Young’s modulus. The addition of chitosan to the filaments improved their ability to crystallize and provide their antimicrobial properties against *Escherichia coli* and *Staphylococcus aureus.*

## 1. Introduction

Fused deposition modeling (FDM) is one of the main additive technologies for fabricating three-dimensional objects from simple to complex architectures using computer-aided design models. FDM is a relatively cost-effective and straightforward 3D printing technique, which relies on the utilization of fairly cheap, commercially available thermoplastics such as polylactic acid (PLA) and acrylonitrile-butadiene-styrene (ABS) in a coil form to fabricate desired objects in a layer-by-layer fashion on a print bed [1]. The main expectation for the use of 3D printing technology lies in the healthcare industry, such as customization of patient-specific necessities from implants to drug dosage [2,3,4]. Despite the wide range of possibilities offered by 3D printing technologies, in future trends, it is expected to play an important role in the biomedical sector: printing of complex organs and heterogenic tissues, printing “real” models for medicine testing, and in situ printing during operation with very high precision [5].

The most popular materials used in the FDM method are thermoplastic polymers, such as acrylonitrile butadiene styrene (ABS), polylactic acid (PLA), polyamide (PA), polycarbonate (PC) [6,7,8,9]. For most of these compounds, it is impossible to use them in biomedical applications, because of the need to meet the following crucial requirements: biocompatibility, biodegradability, proper gelation or crystallization kinetics, diffusion properties related to the porosity of materials (nutrients, oxygen and waste transport), or the ability of cells to proliferate, differentiate, migrate, adhere, and spread in direct contact with the material [10]. There are two alternative ways of adapting the FDM method to printing for this sector.

First, the complete substitution of thermoplastic material with natural polymers with the principle of FDM printing called the micro-extrusion bioprinting with alginate solution [11]. A noticeable drawback of sodium alginate for biomedical applications is the low cell attachment and characteristic spreading of the resultant hydrogels crosslinked by divalent cations, less accuracy (resolution) of the printout in comparison with printouts from thermoplastics, or time-consuming chemical modification of natural polymers to introduce cross-linkable moieties. The second method may be using a biocompatible lactic acid polymer (PLA) as a base raw material doped with natural compounds possessing desirable functional properties [12]. An interesting example of using a natural additive to functionalize the filament can be chitosan.

Chitosan is a polysaccharide composed of randomly distributed β (1→4)-linked *D*-glucosamine and *N*-acetyl-*D*-glucosamine units in a linear polymer structure. It is produced by a deacetylation process of chitin occurring in the exoskeleton of shellfish and insects, as well as in the cell walls of fungi [13]. Due to its biocompatibility, biodegradability, antimicrobial properties, non-toxicity, and film-forming properties, chitosan offers potential in a range of applications [14]. In addition, the biological properties of chitosan as binding to mammalian cells, acceleration of the formation of osteoblast responsible for bone formation, immunoadjuvant, hemostatic or regenerative effect on connective tissue, allow the potential use of chitosan as one of the raw materials in the biomedical industry and regenerative medicine [15]. Recently and most widely described in the scientific literature is the creation of scaffolds using 3D printing techniques to grow cells or release active compounds from them.

The current state of knowledge regarding the use of the technique based on the FDM method with chitosan as a raw material for the production of scaffolding for tissue engineering is the following [16]:

Bergonzi et al. proposed a two-step cryo-printing 3D technique using chitosan hydrogel frozen during printing with the post-printing gelation process with different hydration abilities depending on the composition of the gelation solution [17]. Wang et al. used hydrohybutyl chitosan in the sol–gel transition temperature connected with post-processing gelation in deferent salt solutions to design a system for chondrocytes growth [18]. Li et al. reported a promising strategy to print 3D cells with strong interfacial bonding, where the κ-carrageenan and gelatin methacrylate were found to be the best combination for obtaining scaffolds for mouse myoblasts cells C_2_C_12_. The use of chitosan gave worse results [19]. The works concerning PLA modification with chitosan are mainly focused on chemical functionalization of the PLA surface, often with using cross-linking agents [20,21] and solution mixing and casting procedure [22,23]. Mainly these studies concern modification of thermoplastic polymers using chitosan in forming functional films. But the only work closest to the solution we present are the results of Wu and colleagues [24]. They used PLA and chitosan to create a PLA/CS composite; CS was first made into CS powder by grinding, then mixed into PLA and finally grafted by maleic anhydride (MA) into PLA-g-MA/CS, to increase interfacial adhesion of the blend and enhance the mechanical properties of the PLA/CS composite strip in reference to the neat PLA.

Recently, no work shows how the properties of chitosan itself affect the quality of extruded PLA-based filaments for 3D printing and then obtain objects from them, which is the basis for planning subsequent experiments in obtaining new functional materials. This is particularly important in the case of biological raw materials characterized by high variability: different molecular weights, varying degrees of polydispersity, or deacetylation degree in the case of the chitosan. Our work is an attempt to prove that despite the low interfacial interaction between the PLA and chitosan, it is possible to achieve antimicrobial filaments for 3D printing with the FDM technique, even at low chitosan concentration. We also wanted to prove that remelting the composite during 3D printing does not have a negative impact on the antimicrobial properties of the designed and printed objects.

## 2. Materials and Methods

### 2.1. Materials

Medium molecular weight chitosan polymer (MMW) with 75–85% deacetylation degree (viscosity 200 ÷ 800 cps 1% concentration solution in 1% acetic acid at 25 °C) and KH_2_PO_4_ were obtained from Sigma–Aldrich (Saint Louis, MO, USA). The PLA and PLA derivatives (sPLA) pellets for extrusion were purchased from ORBI-TECH (both processing temperature: 190–225 °C) Leichlingen, Germany). For microbiological tests, the following bacterial strains were used: Gram (−) *E. coli* K-12 PCM 2560 (NCTC 10538) and Gram (+) *S. aureus* PCM 2054 (ATCC 25923) from the Polish Collection of Microorganisms, Ludwik Hirszfeld Institute of Immunology and Experimental Therapy of the Polish Academy of Sciences (Wrocław, Poland). The nutrient agar was purchased from BTL Sp. z o.o. (Łódź, Poland).

### 2.2. Filament Preparation

Table 1 presents a description of the samples used in the experiment, taking into account the mass of chitosan extruded with PLA/sPLA.

A small laboratory extruder with two temperature zones and auto winder (Wellzoom C, Zhenzen, China) was used to obtain the filaments. The molding nozzle diameter was equal to 1.75 mm. Several parameter combinations were tested to obtain filaments of stable diameter dimension, for the production of which the same temperatures of the tank with the pellet’s feeder (zone 1–205 °C) and the molding nozzle (zone 2–190 °C) were used. The filament diameter during extrusion was controlled by the dial indicator. The PLA and sPLA filament was obtained by directly placing the material pellets in the extruder hopper. To modify PLA or sPLA, the mixtures of pellets with chitosan powder were combined in a suitable weight ratio (Radwag PS1200.R2, Radom, Poland) and thoroughly mixed before placing in the extruder.

### 2.3. Filament Characterization

#### 2.3.1. Density

To determine the density (*ρ*) of materials, the filaments were cut into pieces with 100 ± 1 mm lengths and conditioned in a humidity chamber at 25 °C and relative humidity at 50% within 24 h (Memmert HCP 108 GmbH + Co. GK, Schwabach, Germany). Then, each sample was weighed with an accuracy of 0.0001 g (Sartorius 1801, Göttingen, Germany), and the diameter was measured using an electronic micrometer (Satra S-MIC50, Mansfield, UK). The diameter measurement was carried out in three sample locations, and for further calculations, the arithmetic mean of these results was used. The density of the filaments was calculated based on Equation (1).

(1) $$\rho \;=m/\pi r^{2}l,$$

where: *m*—mass of the filament with the length of 100 mm [g], *l*—filament length [cm], *r*—filament radius [cm].

#### 2.3.2. Topography Evaluation

The topography evaluation was carried out using a variable-pressure scanning electron microscopy VP-SEM S3400-N (Hitachi, Hyogo, Japan). The accelerating voltage was 20 kV. The charge compensation was assured through conducting micrographs at 120 Pa and Back-scattered Electron Detector (BSE). Utilization of BSE detector allowed to increase the contrast of local areas varying in the material’s chemistry and/or density. The pictures of filament fragments were taken with a Nikon D7200 camera (Warszawa, Poland).

#### 2.3.3. Fourier-Transform Infrared Spectroscopy (FT-IR) Study

The FT-IR spectra were measured using a spectrometer (Nicolet 8700; Thermo Electron Corp., Waltham, MA, USA) equipped with the GoldenGate (Specac Corp., Oprington, UK) ATR accessory with a single reflection diamond crystal. The temperature of the crystal was maintained at 25.0 ± 0.1 °C by using an automatic temperature controller (Specac Corp., Orpington, GB) coupled with the ATR accessory. In each measurement, 64 scans were collected with a resolution of 4 cm^−1^ and the range of 4000 to 550 cm^−1^. The spectrum of the filament was measured and later subtracted from every measured spectrum as the background. After measuring all FT-IR spectra corresponding to a selected strain and background subtraction, the average spectrum was calculated. The spectrometer was purged with dry nitrogen to diminish the negative influence of water vapor. Spectragryph V. 1.2.10 software (Oberstdorf, Germany) was used to process the obtained spectra.

#### 2.3.4. Mechanical Properties

The mechanical tensile test properties of the filament samples were obtained using a universal testing machine (Instron model 5543, controlled using the “Merlin” software V 4.42., Warszawa, Poland) according to ISO 527:2012 standard.

#### 2.3.5. Thermal Properties

The study of thermal analysis was carried out with a DSC Diamond Perkin Elmer (Norwalk, CT, USA), which was calibrated against the indium standard in a nitrogen atmosphere (flow rate of 20 mL/min). An empty aluminum pan was the reference. The weight of the samples used for the determinations was 10 ± 2 mg. The DCS procedure consisted of three steps. In the first step, samples were heated from 25 to 200 °C with a heating rate of 10 °C/min. Then they were cooled to 25 °C, at a cooling rate of 10 °C/min. In the last step, they were reheated to 200 °C at a heating rate of 10 °C/min. From the DSC thermograms (second run), the effect of chitosan was studied on the thermal properties of the PLA, such as the glass transition temperature (*T*_g_), exothermic crystallization temperature (*T*_c_), endothermic melting temperature (*T*_m_) [25].

#### 2.3.6. Antimicrobial Properties

The evaluation of antimicrobial properties of filaments was made according to the quantitative ASTM E2149 method with slight modification using *E. coli* and *S. aureus* strains. The strains were deep-frozen and stored in CrioBanks; their biochemical features were regularly controlled, and before the test, they were multiplied on nutrient agar. A sample of 1 g of filament/obtained 3D object previously cut into 2 mm fragments was put into a 250 mL conical flask and flooded with 0.3 mM KH_2_PO_4_ solution with bacteria at a density of 3.0 × 10^5^ cfu/mL. Samples in two repetitions and a reference without a sample were incubated for 1 hour at 37 °C (Heidolph Incubator 1000, Merck Sp. z o.o., Warszawa, Poland) with constant shaking at 200 rpm (Heidolph Unimax 1010, Merck Sp. z o.o., Warszawa, Poland). The 1 mL of the suspension was collected from each of the flasks, and the number of bacteria (cfu/mL) was estimated by the method of decimal dilution on nutrient agar. Incubation was made from either dilution onto two parallel plates. The result of the analysis is presented as a reduction in the number of bacteria calculated from the formula:

Reduction [%] = (B − A)/B ×100(2)

or

Reduction [log] = (log B − log A),(3)

where A—cfu of 1 mL in the tested sample after 1 hour of incubation, B—cfu of 1 mL estimated in the starting suspension before the addition of the sample (time 0).

#### 2.3.7. Statistical Analysis of the Data

The presented results are the average values from at least three replications. The data were evaluated by analysis of variance (one-way ANOVA procedure) using the program SigmaPlot 11.0 (Systat Software, Erkrath, Germany), and the differences between the means were determined by Tukey’s multiple tests (*p* < 0.05).

### 2.4. 3D Print Preparation

The print model was downloaded from the free3d.com website as the object file. For the production of the tested samples, a desktop-grade FDM 3D printer Zortrax M200 Plus (Olszytyn, Poland) was fed with obtained PLA and PLA-CHI_3_ filaments with a specified diameter of 1.75 mm. The G-code was made by software Z-Suite v2.11.1 which is part of the 3D printer pack. Printing process parameters were set as high quality and maximum infill, other process parameters were set automatically by the software as an optimal value which company Zortrax recommended for each filament separately.

## 3. Results and Discussion

The fused deposition modeling is the method of 3D printing, in which a continuous filament of material is heated in the nozzle to reach a semi-liquid state and then extruded on the platform (first layer) or on the top of the previously printed layers [26]. The filament has to be thermoplastic because it allows combining subsequent layers of print and solidifying the entire object after reaching room temperature. Using bulk modification, such as copolymerization with other lactone-type monomers, PEG, monomers with functional groups, and blending with other materials, the degradation rates, hydrophilicity, mechanical properties, and surface properties of PLA-type polymers can be significantly changed [27]. Therefore, to preserve the thermoplastic properties of the mixtures of PLA with chitosan, a maximum of 10% (*w/w*) of the latter was used.

### 3.1. Structure of Filaments

The co-extrusion of PLA and chitosan was successful, and it was possible to obtain a filament with a diameter of 1.75 ± 0.03 mm. The results of the filament density measurements are shown in Table 2. The density range of PLA and its derivatives, such as PLLA and PDLA, has been reported as 1.21–1.25 g/mL, 1.24–1.30 g/mL, and 1.25–1.27 g/mL for the amorphous and crystalline form of polymer, respectively [28]. The density consistent with the literature data was obtained only for PLA and PLA-CHI_3_ samples. The 3% (*w/w*) share of chitosan (with a density of 0.35 g/mL) in the filament did not significantly affect its density. However, the 10% (*w/w*) share of chitosan caused a decrease in the density of PLA and sPLA filament by 45% and 25%, respectively.

Values obtained for PLA and sPLA materials marked with the same letters do not differ significantly.

All samples of the sPLA filaments had a higher density than the corresponding PLA filament samples. This may be due to the higher degree of crystallinity of the lactic acid polymer in the sPLA granulate. Moreover, the presence of the high molecular weight plasticizer could provide sPLA filaments greater flexibility. A similar dependence on lowering the filament density with the increase in the contribution of the modifying additive was presented by Daver and colleagues [29]. They created a cork-PLA filament for printing using the FDM technique, which included a 5% (*w/w*) of the cork. The 3D printed composite showed slightly lower tensile mechanical properties, except elongation at break where 3D printed composite was more ductile compared to that of compression molded composites.

**Figure 1:**
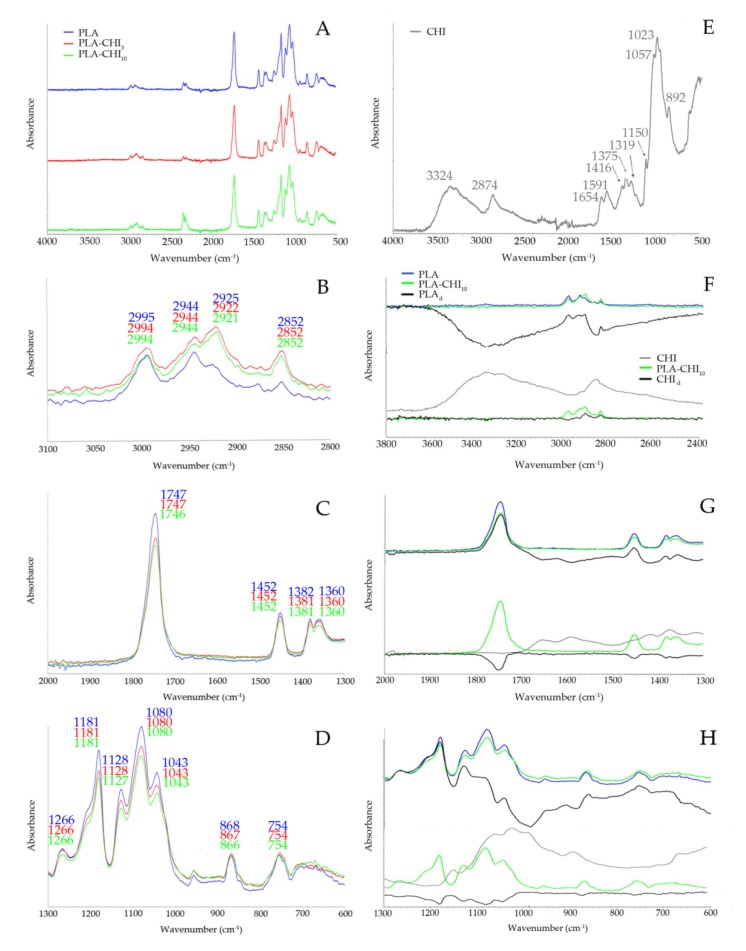
(**A**–**D**) FT-IR spectra of polylactic acid (PLA), PLA-CHI_3_, PLA-CHI_10_, (**E**) FT-IR spectrum of CHI; (**F**–**H**) FT-IR spectra of the PLA, PLA-CHI_10_, and difference spectrum of PLA-CHI_10_ from which the spectrum of chitosan (CHI) was subtracted (PLA_d_) on the top, FT-IR spectra of the CHI, PLA-CHI_10_, and difference spectrum of PLA-CHI_10_ from which the spectrum of PLA was subtracted (CHI_d_) on the bottom. (Different colored lines in B,C and D represent the same substance as A; different colored lines in G,H represent the same substance as F).

Figure 1 shows FT-IR spectra of PLA, PLA-CHI_3_, and PLA-CHI_10_ filaments. The stack spectra showed that the chitosan additive did not have a significant impact on the structure of PLA (Figure 1A). In the range of 3100 to 2800 cm^−1^ in the PLA spectrum, the characteristic stretching frequencies for –CH_3_ asymmetric and –CH_3_ symmetric vibrations at 2995 and 2944 cm^−1^, respectively, were present (Figure 1B). The asymmetric and symmetric bending frequencies for the same group have been identified at 1452 and 1382 cm^−1^, respectively (Figure 1C) [30]. The peaks located at 2925 and 2852 cm^−1^ showed the –CH– asymmetric and the –CH– symmetric stretching vibrations, respectively (Figure 1B) [31]. In Figure 1C, two peaks at 1747 and 1360 cm^−1^ corresponding to the stretching vibration of the ester carbonyl group and –CH group were also identified. The second one used to be sharper for PLA_LMW_ than for PLA_HMW_ due to the differences in chain conformation arranges in each polymer. It is one of the markers confirming that the high molecular weight PLA was used during our studies. The next marker of the PLA molecular mass is the band of carbonyl ester group appears at 1266 cm^−1^, which is stronger for PLA_HMW_ compared to PLA_LMW_ due to the difference in the number of ester links [32]. The next band at 1181cm^−1^ was ascribed to –C–O– stretching bond vibration in the –C–OH– group of PLA. The specific region composed of three characteristic peaks, ascribed to –C–O– stretching vibration in –O–C=O group, was identified at 1128, 1080, and 1043 cm^−1^, respectively [33]. Figure 1E shows the chitosan (CHI) spectrum, which demonstrated a broad band in the range of 3600 to 2700 cm^−1^, attributed to $\upsilon_{\mathrm{NH}\;}$and $\upsilon_{\mathrm{OH}\;}$vibration (3324 cm^−1^), and stretching vibrations of –CH_2_– group at 2874 cm^−1^. The peak at 1654, cm^−1^ corresponds to the amide I band. The second derivative procedure of chitosan spectrum in work of Staroszczyk and colleagues [34] showed that the signal around 1590 cm^−1^ can be related with the $\delta_{\mathrm{NH}}$ vibration of the free –NH_2_ group. Next two peaks at 1416 and 1375 cm^−1^ come from the –CH_2_– group’s vibration area, asymmetrical bending and stretching, respectively. The amide III band located at 1319 cm^−1^ corresponds mainly to the stretching C–N and bending N–H vibration. The saccharide region of the spectrum includes the asymmetric stretching vibration of the C–O–C bridge at 1150 cm^−1^, the skeletal vibrations involving the C–O–C stretching band at 1057 and 1023 cm^−1^ and the last peak in the saccharide region of the spectrum at 892 cm^−1^ corresponding to the $\delta_{\mathrm{CH}}$ vibration of βthe -glycosidic bond [35].

The addition of chitosan to the filaments slightly weakened the intensity of the bands in the range of wave numbers 1800 to 1000 cm^−1^ and increased the intensity of the bands in the range of wave numbers 3000 to 2800 cm^−1^. However, the differential spectra of PLA and chitosan (PLA_d_, CHI_d_) present in Figure 1F–H confirmed that co-extrusion of these two components did not cause the disappearance or appearance of new bands. This means that the components of the mixture did not react with each other and formed only a physical mixture. Small differences in the intensity of the spectra and shifts of no more than 5cm^−1^ indicate conformation changes in the chains of both polymers, related to their most energetically advantageous way of arranging them in a two-component filament.

**Figure 2:**
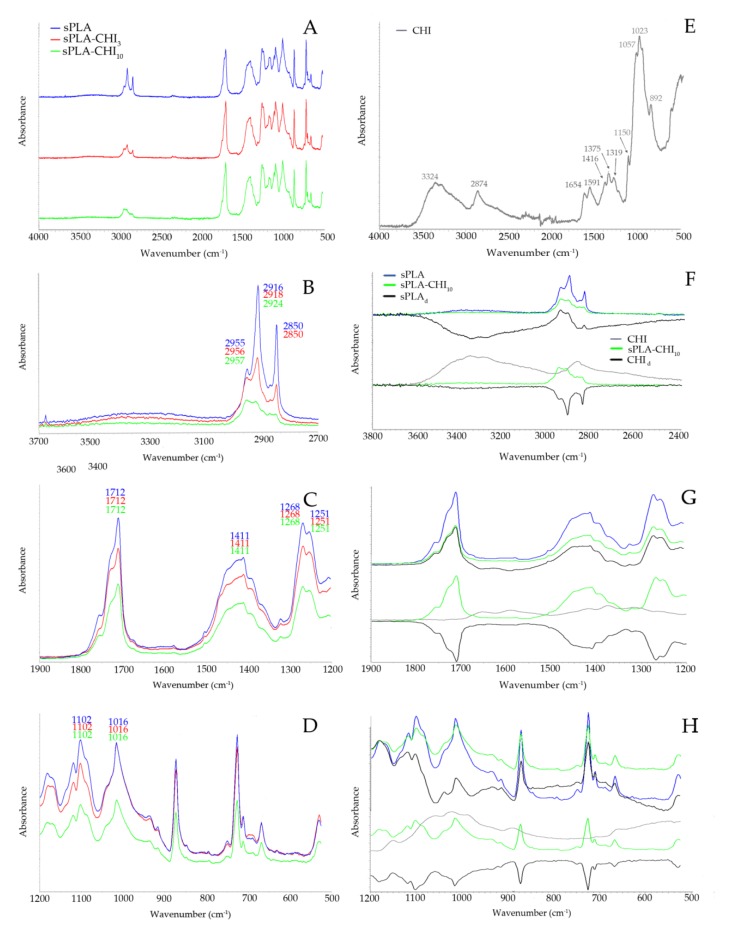
(**A**–**D**) FT-IR spectra of sPLA, sPLA-CHI_3_, sPLA-CHI_10_, (**E**) FT-IR spectrum of CHI; (**F**–**H**) FT-IR spectra of the sPLA, sPLA-CHI_10_, and difference spectrum of sPLA-CHI_10_ from which the spectrum of CHI was substracted (sPLA_d_) on the top, FT-IR spectra of the CHI, sPLA-CHI_10_, and difference spectrum of sPLA-CHI_10_ from which the spectrum of sPLA was substracted (CHI_d_) on the bottom. ((Different colored lines in B,C and D represent the same substance as A; different colored lines in G,H represent the same substance as F).

Figure 2 shows FT-IR spectra of sPLA, sPLA-CHI_3_, and sPLA-CHI_10_ filaments. The stack spectra indicate a clear difference in the range of wave numbers 3100 to 2800 cm^−1^ (Figure 2A). In Figure 2B, the wide band in the range of wave numbers 3500 to 3100 cm^−1^ can be observed, whose intensity decreased with increasing concentration of chitosan in the filament. Such band did not appear in the PLA spectra (Figure 1), which may indicate the stretch vibrations of the –OH groups of the plasticizer present in the sPLA granulate, for example, amorphous vinyl acetate units comes from EVA polymer [36]. The addition of chitosan also caused the reduction in the intensity of the bands corresponding to C–H stretching vibrations of –CH_3_ (2955 cm^−1^) and –CH– groups (2916 cm^−1^, 2850 cm^−1^). The occurrence of the C=O stretching band of the carbonyl group in sPLA at 1712 cm^−1^ (Figure 2C), the wave number of approximately 40 cm^−1^ lower than for the PLA may indicate the vibration in sPLA higher number of groups ester carbonyl group [37]. The irregular band at the 1411 cm^−1^ wave number may be the result of an overlap of the three bands of PLA: 1452, 1382, and 1366 cm^−1^ (Figure 1C) and absorption peak attributed to the contributions from both VA and ethylene (–CH_2_–) units [37]. The peak at 1268 and 1251 cm^−1^ is assigned to –C=O bending from PLA and plasticizer, respectively (Figure 2C) [36]. The spectra also showed the characteristic bands ascribed to –C–O– stretching vibration in –O–C=O group at the 1200–1000 cm^−1^ region (Figure 1D). The band at 1016 cm^−1^ can correspond to the symmetric stretching vibration of the C–O–C band of EVA [37]. The addition of chitosan in filaments weakened the intensity of the bands in the whole measuring range (Figure 2B–D). The differential spectra of sPLA and chitosan (sPLA_d_, CHI_d_) present in Figure 2F–H, confirmed that co-extrusion of these two components did not cause the disappearance or appearance of new bands. Therefore, it can be concluded that sPLA, in spite of the presence of a plasticizing compound, also formed only physical mixture during co-extrusion with chitosan.

**Figure 3:**
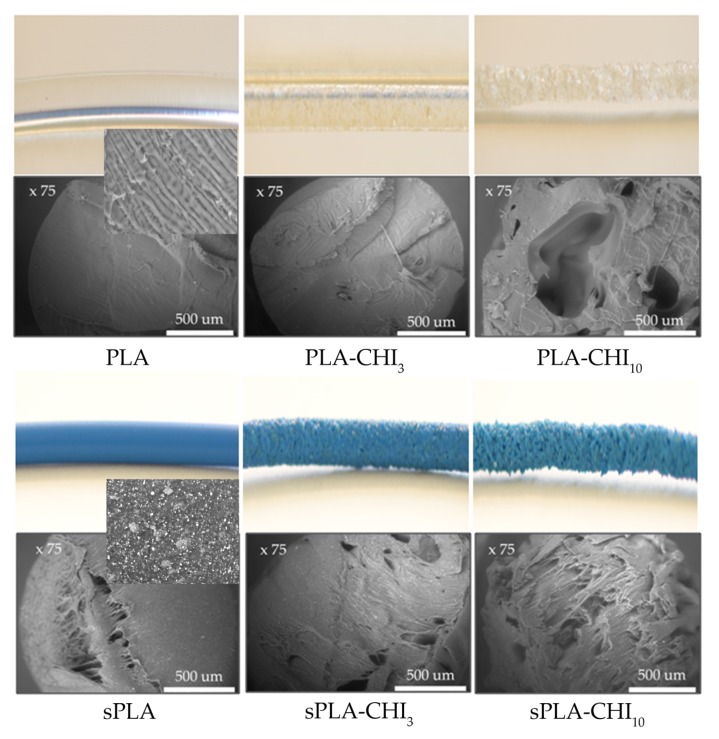
Comparison of the optical and scanning images of the obtained filaments. In the inset, magnified areas, revealing the uniform distribution of pigment/plasticizer in sPLA sample.

The change in the density of materials doped with chitosan has also been confirmed based on images and SEM micrographs (Figure 3).

The PLA and sPLA filaments were homogeneous and had smooth surfaces. The 3% addition of chitosan did not cause a significant difference in the microstructure of these samples. In turn, the PLA-CHI_10_ filament was porous and characterized by an irregular, rough surface. Thus, its density was the smallest. A similar pattern was observed for sPLA, sPLA-CHI_3_, sPLA-CHI_10_ samples. The BSE analysis of sPLA filaments showed the presence of a homogeneously dispersed plasticizer or pigment, providing a blue color of granulate, also confirmed by FT-IR studies.

### 3.2. Mechanical Properties

Due to the inability to use filaments containing a 10% chitosan additive (PLA-CHI_10_ and sPLA-CHI_10_) in the FDM printing process, the results regarding the effect of chitosan additive on mechanical properties were determined by directly testing the filaments and not printed therefrom the 3D objects. From the tensile testing result data, mechanical property information was extracted by plotting the stress-strain curves. Table 3 demonstrates the list of average diameters, tensile strength, elongation at break, and Young’s modulus. Co-extrusion of PLA or sPLA with chitosan allowed obtaining filaments of the same diameter. The PLA filament was characterized by tensile strength and modulus of elasticity, similar to those demonstrated by other authors [38,39]. The 3% (*w/w*) addition of chitosan in the PLA-CHI_3_ filament did not change the mechanical properties apart from the elongation at break, which was 80% lower compared to the PLA. The 10% (*w/w*) addition of chitosan in PLA-CHI_10_ sample significantly degraded filament properties, for which tensile stress, elongation, and modulus of elasticity were 58%, 91%, and 28% lower than for PLA, respectively. The sPLA was characterized by significantly higher elongation at break, lower tensile strength, and lower Young’s modulus compared to the PLA sample. The 3% (*w/w*) addition of chitosan in the sPLA-CHI_3_ sample resulted in 51% and 96% reduction of tensile strength and elongation at break, respectively. Increasing the addition of chitosan up to 10% (*w/w*) in the sPLA sample further deteriorated the mechanical properties of the filaments. It also caused a decrease in the value of Young’s modulus. For the PLA and the sPLA filaments, with increasing chitosan content, a decrease in tensile strength, elongation at break, and Young’s modulus were observed. Moreover, the relationship between decreasing Young’s modulus with a decreasing density of materials has been presented by Vanleene and colleagues [40]. Based on Young’s modulus–density and Young’s modulus–tensile strength charts available in Material Data Book [41], all obtained sPLA filaments can be classified as elastomeric materials, while almost all obtained PLA filaments showed properties characteristic a of thermoplastic polymer. The exception was the PLA-CHI_10_, which based on Young’s modulus–density chart, was classified as a natural material with properties similar to the typical wood. Wu et al. in their work recorded that tensile strength of neat PLA (44.3 MPa) decreased to 42.6 MPa following grafting with maleic anhydride (MA), and the tensile strength of PLA/chitosan composites decreased markedly with increasing chitosan content attributed to the poor dispersion of chitosan in the PLA matrix. However, the PLA/chitosan composites exhibited an increase in tensile strength at failure as the filler of the chitosan content reached saturation at content exceeding 10 wt %. This behavior was attributed to improved dispersion and covalent bonding of the chitosan in the PLA-g-MA matrix resulting from the formation of branched or cross-linked macromolecules. The tensile strength of the PLA-g-MA/chitosan composites was ca. 7–30 MPa greater than that of the PLA/chitosan composites [24]. 

### 3.3. Thermal Properties

**Figure 4:**
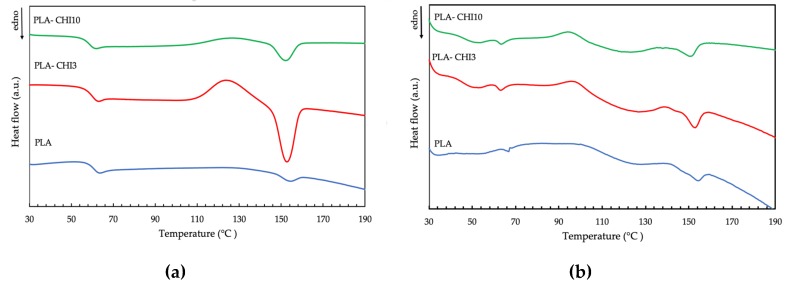
Differential scanning calorimetry (DSC) thermograms of (**a**) PLA, PLA-CHI_3_, and PLA-CHI_10_ samples; (**b**) sPLA, sPLA-CHI_3_, and sPLA-CHI_10_ filaments. (“Endo” indicates the direction of endothermic transformations during sample heating).

Differential scanning calorimetry (DSC) measures the amount of heat energy absorbed or released when a material is heated or cooled. For polymeric materials, which undergo important property changes near thermal transition, the DSC is a very useful technique to study the glass transition temperature, crystallization temperature, and melting behavior. The DSC curves of the neat PLA, neat sPLA, and their composites with chitosan are presented in Figure 4, and determined thermal characteristics are given in Table 4. All characteristic temperatures of PLA samples (*T*_g_, *T*_c_, *T*_m_) were similar to those obtained for thermal treated PLA in Carrasco and colleagues’ work [42]. The neat PLA filament showed two small endothermic peaks. The first was the glass-transition temperature (*T*_g_) at 63.9 °C, and the second was melting temperature (*T*_m_) at 154.5 °C, respectively. The addition of chitosan into the PLA polymer led to a minor decrease in these two temperatures. This is because the chitosan increases the free volume and flexibility of polymeric chains [43]. The addition of chitosan to the filament improved its ability to crystallize. Moreover, the crystallization temperature of the sPLA-CHI_3_ and the sPLA-CHI_10_ filaments were lower than for the neat sPLA sample (Table 4), which indicates that the chitosan can act as a nucleating agent promoting crystallization.

The addition of chitosan in both types of PLA samples resulted in the lowering of the melting temperature. The obtained filaments were a mixture of the amorphous and crystalline phases. After melting, the two phases mix to one phase, which after cooling, crystallizes (without de-mixing), forming one “impure” solid phase. Mucha and Królikowski [44] found that an organic filler, such as chitosan powder, forms the amorphous inclusion in the composites. On reheating, this single phase can melt at a lower temperature because of the adsorbed “impurities” of the former amorph phase, which causes a melting point depression. The changes in the crystallization and melting temperatures associated with the presence of chitosan in the filaments would not significantly affect the printing conditions, since the largest temperature differences recorded between the sPLA and sPLA-CHI_10_ samples for *T*_g_, *T*_c_, *T*_m_ were 2.3, 4.8, and 3.5 °C, respectively.

### 3.4. Antimicrobial Properties

To evaluate the antimicrobial properties of the filaments the ASTM: E2149 method was used, which is designed to measure the antimicrobial activity of non-leaching (non-water soluble) antimicrobials surfaces made of plastic, rubber, silicone, and treated fabric material and is one method to test an irregularly shaped antimicrobial object, such as a thread, powder, 3D molded plastic. The results presented in Table 5 and Table 6 indicate that the control materials (PLA and sPLA) did not reduce the number of *E. coli* and *S. aureus*.

In these samples, even an increase in the number of microorganisms was noted. The obtained results seem to be correct because PLA does not show antimicrobial activity. The only effect of limiting the growth of microorganisms by pure PLA has been demonstrated in a coating experiment [45]. However, it was related to the poor oxygen permeability of the PLA film [38,46]. With the increase in the concentration of chitosan in the filament, the average reduction in the number of *S. aureus* bacteria increased. The highest value was achieved for PLA filament with a 10% addition of chitosan (Table 5). A similar pattern was observed regarding *E. coli* bacteria (Table 6). It means that a greater antimicrobial effect of the filaments was obtained with respect to the Gram-positive strain. The 3D prints showed virtually the same antimicrobial properties as the filaments from which they were printed. It applies to both tested bacteria strains. This means that re-melting the PLA-chitosan material has no significant effect on the inhibition of the material’s antimicrobial activity. The antimicrobial activity was due to the presence of chitosan and depends on the degree of its deacetylation, molecular weight, concentration in solution, pH, and ionic strength of the solution [14]. According to Goi and colleagues, there is a lack of conclusive data on whether the chitosan has higher activity on Gram-positive or on Gram-negative bacteria [47]. On both strains, chitosan seems to act differently, though in both cases satisfactorily. The main reason for the antimicrobial activity of chitosan is the interaction of its positive charged chains with anionic components of microorganisms—lipopolysaccharides (Gram-negative bacteria) and teichoic acids (Gram-positive bacteria) that cause bacteria cell lysis. However, this mechanism takes place when the amino groups of the polymer are protonated: pH < 6 [48]. It has been concluded that for neutral or alkaline media, the cationic nature of chitosan can no longer explain its antibacterial activity. In this case, the strong coordination capability of –NH_2_ groups in the chitosan chain might be one possible mechanism [49,50]. Considering the test conditions and comparing them with the conditions in other, most frequently used standards [51,52], it can be concluded that the addition of chitosan allows obtaining at least bacteriostatic filaments. For comparison, antimicrobial activity tests performed for the *E. coli* strain in Wu et al. studies showed the PLA/chitosan or PLA-g-MA/chitosan suppressed the growth of *E. coli*; furthermore, all of the samples containing PLA-g-MA/CS exhibited a higher degree of bacterial suppression than the corresponding samples of PLA/CS. The antibacterial effect on bacteria was enhanced in PLA-g-MA/CS because the PLA-g-MA is stabilized in a fixed orientation by CS, resulting in bacteria death due to increasing effective concentration of CS to bacteria or contacting time elongation to bacteria [24].

### 3.5. Sample Print Effect

**Figure 5:**
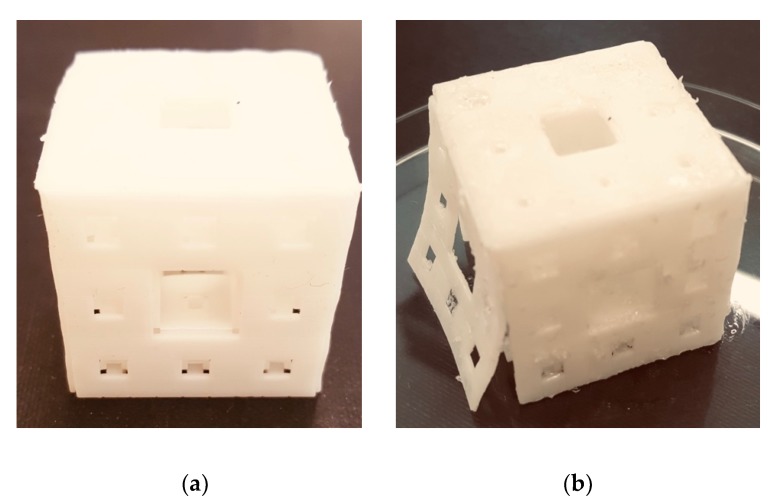
Sample 3D print using (**a**) PLA filament, (**b**) PLA-CHI_3_ filament.

An example of 3D printing using PLA and PLA-CHI_3_ filament is shown in Figure 5. The obtained printout is a form of a perforated cube. The cube obtained from PLA is of good quality, and its edges are sharp and clear. All cube walls are permanently connected to each other. A 3% addition of chitosan caused the printout to be less accurate (blur effect). On its walls were visible points indicating the heterogeneity of the material. Moreover, the same addition of chitosan in PLA caused an increase in shrinkage of this thermoplastic material during printing, as indicated by one of the cube walls detaching from the entire body. Obtaining a print from a filament containing 10% chitosan was impossible. For prints obtained from PLA and PLA-CHI_3_ filaments, a single compression test was carried out using the Instron universal testing machine (2.3.4.) until 50% deformation of the object relative to its initial height was achieved. The compression speed was 100 mm/min. It turned out that the object containing a 3% addition of chitosan, despite structural defects (Figure 5), was 15% harder than the object made of neat PLA (113 ± 7 and 89 ± 4 MPa, respectively; *n* = 5, *p* <0.05).

## 4. Conclusions

Co-extrusion of the PLA and sPLA granulated with chitosan seemed to be a simple and high-temperature method of producing filaments for 3D FDM printing when the amount of chitosan concentration is low (below 3% *w/w*). For the use of the chitosan additive above 10% (*w/w*), it was difficult to obtain homogeneous materials. Despite the low interfacial interaction between the PLA and chitosan, it was possible to achieve antimicrobial filaments for 3D printing with the FDM technique, even at low chitosan concentration, and this activity can be described as bacteriostatic. To obtain bactericidal activity, chemical modification of chitosan or PLA was necessary so that their hydrophobicity/hydrophilicity should be closer to each other. We also proved that re-melting the filament during 3D printing did not have a negative impact on the antimicrobial properties of the designed and printed objects (PLA-CHI_3_). Thus, this kind of material may have the potential for the biomedical sector in manufacturing bacteriostatic products.

## Figures and Tables

**Table 1:** Description of the samples used in the experiment.

Type of Filament	Description
PLA	Polylactic acid
PLA-CHI_3_	Polylactic acid modified with 3% (*w/w*) addition of chitosan
PLA-CHI_10_	Polylactic acid modified with 10% (*w/w*) addition of chitosan
sPLA	Soft form of polylactic acid
sPLA-CHI_3_	Soft form of polylactic acid modified with 3% (*w/w*) addition of chitosan
sPLA-CHI_10_	Soft form of polylactic acid modified with 10% (*w/w*) addition of chitosan

**Table 2:** Density of the obtained filaments. Results are a mean ± SD of three independent experiments; n = 3, *p* < 0.05.

Type of Filament	Density [g/mL]
PLA	1.28 ± 0.006 ^a^
PLA-CHI_3_	1.23 ± 0.025 ^a^
PLA-CHI_10_	0.70 ± 0.008 ^b^
sPLA	1.47 ± 0.044 ^A^
sPLA-CHI_3_	1.35 ± 0.069 ^A^
sPLA-CHI_10_	1.11 ± 0.023 ^B^

Values marked with the same letters do not differ significantly: (small letters) against the PLA control sample, (big letters) against the sPLA control sample.

**Table 3:** The comparison of the mechanical properties of the obtained filaments. The values in individual columns marked with the same letters do not differ significantly (*n* = 7; *p* < 0.05).

Type of Filament	Diameter [mm]	Tensile Strenght [MPa]	Elongation at Break [%]	Young’s Modulus [MPa]
PLA	1.74 ± 0.02 ^a^	58.9 ± 1.4 ^a^	32.8 ± 2.2 ^a^	1987 ± 72 ^a^
PLA-CHI_3_	1.76 ± 0.09 ^a^	61.4 ± 0.6 ^a^	6.3 ± 0.6 ^b^	2018 ± 102 ^a^
PLA-CHI_10_	1.77 ± 0.05 ^a^	24.9 ± 1.4 ^b^	2.9 ± 0.4 ^c^	1428 ± 85 ^b^
sPLA	1.75 ± 0.01 ^A^	17.0 ± 0.3 ^A^	572.2 ± 50.3 ^A^	16.4 ± 2.1 ^A^
sPLA-CHI_3_	1.77 ± 0.03 ^A^	8.7 ± 0.4 ^B^	25.3 ± 2.83 ^B^	15.9 ± 1.7 ^A^
sPLA-CHI_10_	1.75 ± 0.02 ^A^	7.6 ± 0.3 ^C^	9.1 ± 0.75 ^C^	3.8 ± 0.4 ^B^

Values marked with the same letters do not differ significantly: (small letters) against the PLA control sample, (big letters) against the sPLA control sample.

**Table 4:** The characteristic thermal transition temperatures of the obtained filaments.

Type of Filament	*T*_g_ [°C]	*T*_c_ [°C]	*T*_m_ [°C]
PLA	63.9	-	154.1
PLA-CHI_3_	63.0	123.2	152.6
PLA-CHI_10_	61.9	125.6	152.0
sPLA	66.7	99.9	154.5
sPLA-CHI_3_	63.1	95.5	152.8
sPLA-CHI_10_	64.4	95.1	151.0

**Table 5:** The antimicrobial activity against *S*. *aureus* of the obtained filaments and prints estimated according to Standard ASTM: E2149.

Type of Filament	cfu/mL	Average Reduction [%]	Average Reduction [log]
Inoculum	5.36 · 10^5^	-	-
PLA *	7.62·10^5^ [6.45·10^5^]	−32.16 [−18.41]	−0.15 [−0.08]
PLA-CHI_3_ *	3.23·10^5^ [3.08·10^5^]	39.74 [41.27]	0.22 [0.24]
PLA-CHI_10_	7.01·10^4^	86.92	0.88
sPLA	6.99·10^5^	−30.41	−0.12
sPLA-CHI_3_	3.54·10^5^	33.96	0.18
sPLA-CHI_10_	2.87·10^5^	46.46	0.27

*The values in square brackets refer to the activity of the materials in the form of a 3D print from the obtained filaments.

**Table 6:** The antimicrobial activity against *E.coli* of the obtained filaments and prints estimated according to Standard ASTM: E2149.

Type of Filament	cfu/mL	Average Reduction [%]	Average Reduction [log]
Inoculum	4.23 · 10^5^	-	-
PLA *	4.96·10^5^ [5.04·10^5^]	−17.26 [−18.41]	−0.07 [−0.08]
PLA-CHI_3_ *	3.84·10^5^ [3.75·10^5^]	9.22 [12.06]	0.04 [0.05]
PLA-CHI_10_	2.64·10^5^	37.59	0.20
sPLA	4.74·10^5^	−12.06	0.05
sPLA-CHI_3_	3.99·10^5^	5.67	0.03
sPLA-CHI_10_	3.35·10^5^	20.80	0.10

* The values in square brackets refer to the activity of the materials in the form of a 3D print from the obtained filaments.
